# Soluble Axl is an accurate biomarker of cirrhosis and hepatocellular carcinoma development: results from a large scale multicenter analysis

**DOI:** 10.18632/oncotarget.17598

**Published:** 2017-05-03

**Authors:** Mirko Dengler, Katharina Staufer, Heidemarie Huber, Rudolf Stauber, Heike Bantel, Karl Heinz Weiss, Patrick Starlinger, Hannelore Pock, Petra Klöters-Plachky, Daniel N. Gotthardt, Peter Rauch, Carolin Lackner, Judith Stift, Christine Brostjan, Thomas Gruenberger, Takashi Kumada, Hidenori Toyoda, Toshifumi Tada, Thomas S. Weiss, Michael Trauner, Wolfgang Mikulits

**Affiliations:** ^1^ Department of Medicine I, Institute of Cancer Research, Comprehensive Cancer Center Vienna, Medical University of Vienna, Vienna, Austria; ^2^ Department of Surgery, Division of Transplantation, Medical University of Vienna, Vienna, Austria; ^3^ Division of Gastroenterology and Hepatology, Department of Internal Medicine, Medical University of Graz, Graz, Austria; ^4^ Department of Gastroenterology, Hepatology and Endocrinology, Hannover Medical School, Hannover, Germany; ^5^ University Hospital Heidelberg, Heidelberg, Germany; ^6^ Department of Surgery, Division of General Surgery, Medical University of Vienna, Vienna, Austria; ^7^ Candor Bioscience GmbH, Wangen im Allgäu, Germany; ^8^ Institute of Pathology, Medical University of Graz, Graz, Austria; ^9^ Clinical Institute of Pathology, Medical University of Vienna, Vienna, Austria; ^10^ Department of Gastroenterology, Ogaki Municipal Hospital, Ogaki, Japan; ^11^ Center for Liver Cell Research, Children's University Hospital (KUNO), University of Regensburg Hospital, Regensburg, Germany; ^12^ Department of Internal Medicine III, Division of Gastroenterology and Hepatology, Medical University of Vienna, Vienna, Austria

**Keywords:** soluble Axl, biomarker, fibrosis, cirrhosis, hepatocellular carcinoma

## Abstract

Patients with chronic liver disease (CLD) and cirrhosis are at high risk for hepatocellular carcinoma (HCC). Current diagnostic tools for HCC detection include imaging techniques and serum biomarkers such as α-fetoprotein (AFP). Yet, these methods are limited in sensitivity and specificity to accurately detect early HCC. Here we focused on the potential of soluble Axl (sAxl) as a biomarker in CLD patients by analyzing serum samples of 1067 patients and healthy controls from centers in Europe and Asia. We show that serum concentrations of sAxl were significantly increased at early (82.57 ng/mL) and later stages of HCC (114.50 ng/mL) as compared to healthy controls (40.15 ng/mL). Notably, no elevated sAxl levels were detected in patients with CLD including chronic viral hepatitis, autoimmune hepatitis, cholestatic liver disease, or non-alcoholic fatty liver disease versus healthy controls. Furthermore, sAxl did not rise in liver adenomas or cholangiocarcinoma (CCA). Yet, patients with advanced fibrosis (F3) or cirrhosis (F4) showed enhanced sAxl concentrations (F3: 54.67 ng/mL; F4: 94.74 ng/mL). Hepatic myofibroblasts exhibited an increased release of sAxl, suggesting that elevated sAxl levels arise from these cells during fibrosis. Receiver operating characteristic curve analysis of sAxl displayed a strongly increased sensitivity and specificity to detect both cirrhosis (80.8%/92.0%) and HCC (83.3%/86.7%) with an area under the curve of 0.935/0.903 as compared to AFP. In conclusion, sAxl shows high diagnostic accuracy at early stage HCC as well as cirrhosis, thereby outperforming AFP. Importantly, sAxl remains normal in most common CLDs, liver adenomas and CCA.

## INTRODUCTION

Hepatocellular carcinoma (HCC) is the most common liver malignancy accounting for about 5% of all cancer cases worldwide and represents the third most common cause of cancer related mortality [1, 2]. Globally, the majority of HCC cases is diagnosed at advanced stages, causing a median survival between less than one and up to four years, depending on the country [3]. However, patients diagnosed at an early stage of tumor development show a favorable five-year survival rate of 70% subsequent to curative treatment strategies such as resection or liver transplantation [4–6].

Notably, about 80% of all HCC cases develop from cirrhosis [1]. Thus, thorough surveillance of this at risk population by ultrasound (US) is mandatory [7]. Yet, an important limitation of this standard method is its poor sensitivity of 63% for detection of early stage HCC and its strong dependence on operator experience [8]. In addition to US, the still most widely used biomarker is α-fetoprotein (AFP), providing a sensitivity between 25% and 65% [8, 9].

Due to these moderate performances of current diagnostic tools, several new biomarkers were proposed. These include lectin bound AFP (AFP-L3%), Dickkopf-1 (DKK1) and des-γ-carboxyprothrombin (DCP). Among these three markers, several studies describe DKK1 as the most sensitive marker to detect HCC, but overall these reports are contradictory [10–13]. Combining AFP with other markers such as AFP-L3%, DCP or DKK1 may lead to improved diagnostic performance [13, 14]. Nevertheless, sensitive and reliable biomarkers for the detection of early HCC and surveillance of patients with chronic liver diseases (CLDs) at high risk for HCC have not been generally established in clinical routine so far [15].

The receptor tyrosine kinase Axl belongs to the TAM receptor family, together with Tyro3 and Mer. Axl dimerization and downstream signaling is induced by the binding of its vitamin-K dependent ligand growth arrest-specific protein 6 to the extracellular domain (ECD) [16]. Axl can be proteolytically cleaved resulting in the release of the ECD termed soluble Axl (sAxl), which can be detected in serum [17]. Elevated levels of sAxl have been observed in several diseases including aortic aneurysm, systemic lupus erythematosus, rheumatoid arthritis, and preeclampsia [18–22]. Recently, we demonstrated an important role of sAxl in HCC [23, 24]. We could show that serum concentrations of sAxl are increased at each stage of HCC including early HCC in contrast to ovarian, breast and colorectal carcinoma (CRC) including CRC-derived liver metastasis. Furthermore, sAxl was significantly higher in HCC patients than in patients with liver cirrhosis only, suggesting that sAxl is a promising diagnostic biomarker for routine clinical use.

In the present large scale multicenter study, we analyzed sAxl levels in sera from 1067 participants suffering from HCC in cirrhotic and non-cirrhotic patients, cholangiocarcinoma (CCA), liver adenoma, and various CLDs with zero to advanced liver fibrosis and cirrhosis. Importantly, this setting allowed us to assess the diagnostic performance of sAxl for HCC in comparison to other solid liver tumors and liver diseases. Furthermore, we were able to determine the accuracy of sAxl in mild to advanced stages of CLD, demonstrating the potential diagnostic value of sAxl for routine clinical use in surveillance of patients at high risk for HCC.

## RESULTS

### High diagnostic value of sAxl for HCC

**Figure 1:**
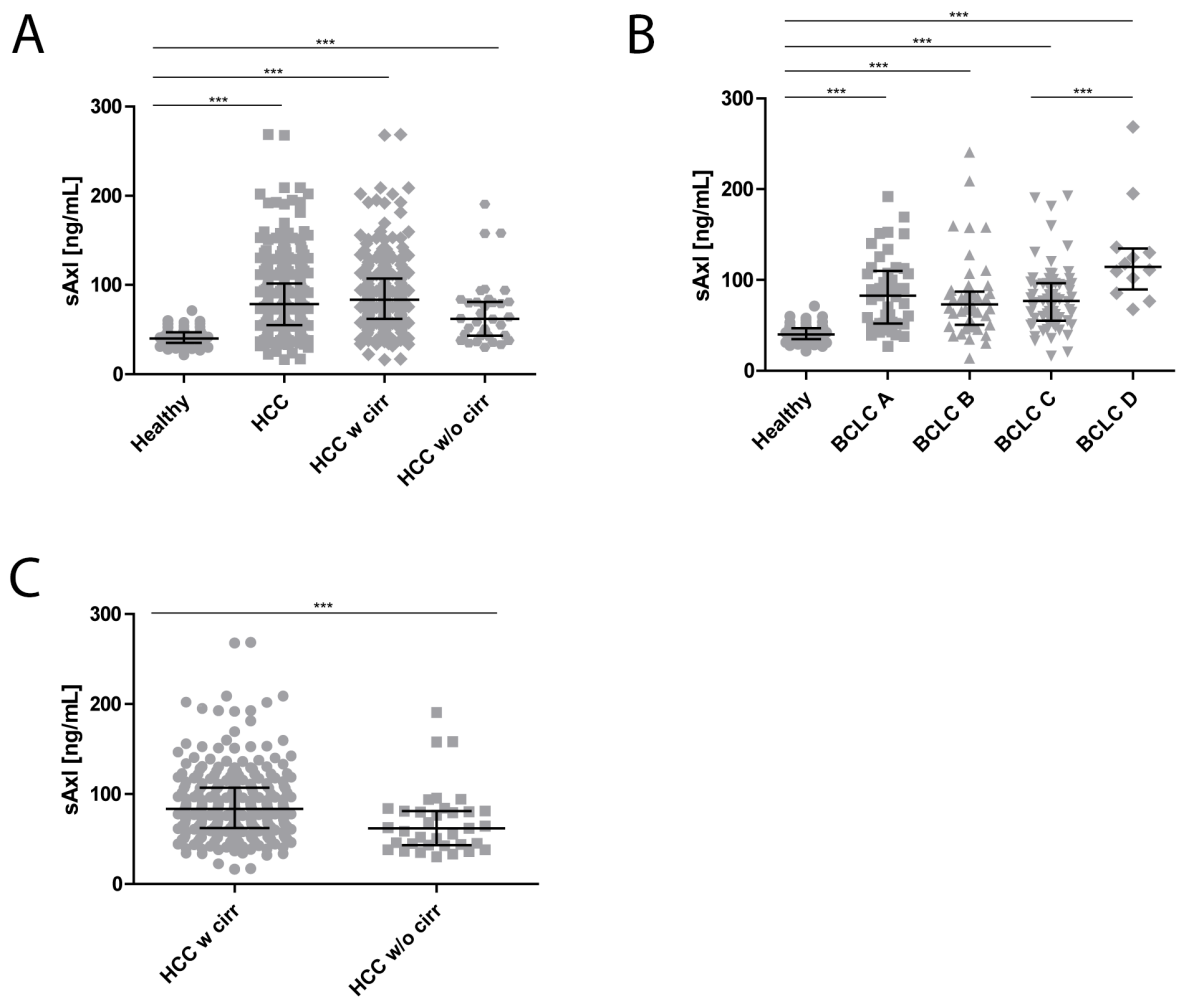
sAxl levels in HCC patients **(A)** sAxl serum concentrations in healthy controls (*n* = 75) compared to all HCC patients (*n* = 347) and in HCC patients either with cirrhosis (*n* = 272) or without cirrhosis (*n* = 35). **(B)** Analysis of sAxl serum concentrations in healthy controls (*n* = 75), early HCC (BCLC A: *n* = 45), intermediate HCC (BCLC B: *n* = 45), advanced HCC (BCLC C: *n* = 67) and end-stage HCC (BCLC D: *n* = 12). **(C)** Statistical comparison between HCC patients with cirrhosis and HCC patients without cirrhosis. Serum samples were diluted with LowCross-buffer^®^ (Candor, Germany) 1:200 and analyzed for sAxl levels by ELISA. Horizontal bars indicate median levels with interquartile ranges. Statistical significances of the differences between groups were evaluated with Mann-Whitney U test. Ns: not significant. *** *p* ≤ 0.001.

We assessed serum levels of sAxl by ELISA in 347 HCC patients and 75 healthy controls from different centers and etiologies (Supplementary Figure 1, Tables 1 and 2). HCC patients were grouped according to liver disease etiology, fibrosis grades, presence or absence of cirrhosis, and BCLC stage (Tables 1 and 2). All HCC patients showed significantly increased median sAxl levels (78.69 ng/mL) in comparison to healthy controls (40.15 ng/mL), independent of the presence of cirrhosis, i.e. 83.37 ng/mL with cirrhosis versus 61.92 ng/mL without cirrhosis (Figure 1A, Table 2). Additional stratification of HCC patients according to BCLC criteria revealed enhanced sAxl levels in early (82.57 ng/mL) but also in intermediate (73.46 ng/mL), advanced (77.08 ng/mL) and end-stage HCC (114.50 ng/mL) as compared to healthy controls (Figure 1B, Table 2). The increase in BCLC D was significant when compared to BCLC A (p = 0.0106), BCLC B (p = 0.0012) and BCLC C (p = 0.0005). Interestingly, HCC patients with cirrhosis had significantly higher sAxl levels in comparison to HCC patients without cirrhosis (Figure 1C, Table 2). From these data we concluded that sAxl accurately detects HCC without cirrhosis as well as HCC in the background of cirrhosis, the latter with higher levels.

**Table 1:** Demographic and clinicopathological attributes of HCC study population

Variable		Number of cases	%	Valid %
**Age (years)**				
Valid	< 55	26	7.5	8.2
	≥ 55	293	84.4	91.8
Missing		28	8.1	
Total		347	100.0	100.0
**Sex**				
Valid	Male	193	55.6	82.8
	Female	40	11.5	17.2
Missing		114	32.9	
Total		347	100.0	100.0
**HBV Status**				
Valid	Negative	133	38.3	88.1
	Positive	18	5.2	11.9
Missing		196	56.5	
Total		347	100.0	100.0
**HCV Status**				
Valid	Negative	113	32.6	74.8
	Positive	38	11.0	25.2
Missing		196	56.5	
Total		347	100.0	100.0
**Cirrhosis**				
Valid	Negative	35	10.1	11.4
	Positive	272	78.4	88.6
Missing		40	11.5	
Total		347	100.0	100.0
**Vascular invasion**				
Valid	Negative	76	21.9	36.2
	Positive	134	38.6	63.8
Missing		137	39.5	
Total		347	100.0	100.0
**Lymph node metastasis**				
Valid	Negative	174	50.1	91.6
	Positive	16	4.6	8.4
Missing		157	45.2	
Total		347	100.0	100.0

HBV: hepatitis B virus; HCV: hepatitis C virus.

**Table 2:** sAxl and AFP serum concentrations of the study population

	Number of cases	Median (IQR) ng/mL
**sAxl**	1067	
Healthy controls	75	40.15 (35.22 - 46.85)
Tumors patients:	402	
All HCC	347*	78.69 (55.09 - 101.5)
HCC with cirrhosis	272	83.37 (62.13 - 107.1)
HCC w/o cirrhosis	35	61.92 (43.13 - 81.09)
BCLC A	45	82.57 (52.13 - 110.2)
BCLC B	45	73.46 (50.59 - 87.24)
BCLC C	67	77.08 (55.09 - 96.33)
BCLC D	12	114.50 (89.69 - 134.6)
CCA	44	41.02 (33.89 - 48.11)
Adenoma	11	31.41 (25.95 - 36.57)
Chronic liver diseases	400	
Fibrosis stages:		
F0	54	40.70 (35.56 - 51.98)
F1	75	46.20 (38.64 - 55.07)
F2	80	46.76 (40.37 - 60.06)
F3	36	54.67 (43.45 - 74.92)
F4 (cirrhosis w/o HCC)	155	94.74 (66.38 - 132.5)
Liver disease etiology:	190	
Viral hepatitis	26	47.87 (42.30 - 61.75)
AIH	18	48.40 (36.16 - 84.20)
Cholestatic liver disease	30	40.97 (36.01 - 51.06)
NAFLD	116	43.15 (37.72 - 53.85)
**AFP**	326	
Healthy controls	28	2.60 (1.500 - 3.475)
All HCC	274	12.90 (5.275 - 173.3)
HCC with cirrhosis	194	11.50 (0 - 49.33)
HCC w/o cirrhosis	28	23.60 (3.900 - 4743)
F4 (cirrhosis w/o HCC)	24	4.65 (3.025 - 6.650)

*347 HCC samples in total, including information for dedicated numbers of cirrhosis status and BCLC stages. Respective clinical data were not available for all 347 HCC cases.

HCC: hepatocellular carcinoma; BCLC: Barcelona clinic liver cancer staging; CCA: cholangiocellular carcinoma; AIH: autoimmune hepatitis; NAFLD: non-alcoholic fatty liver disease; AFP: ?-fetoprotein; IQR: interquartile range.

### Detection of advanced fibrosis and cirrhosis by sAxl independently of liver disease etiology

**Figure 2:**
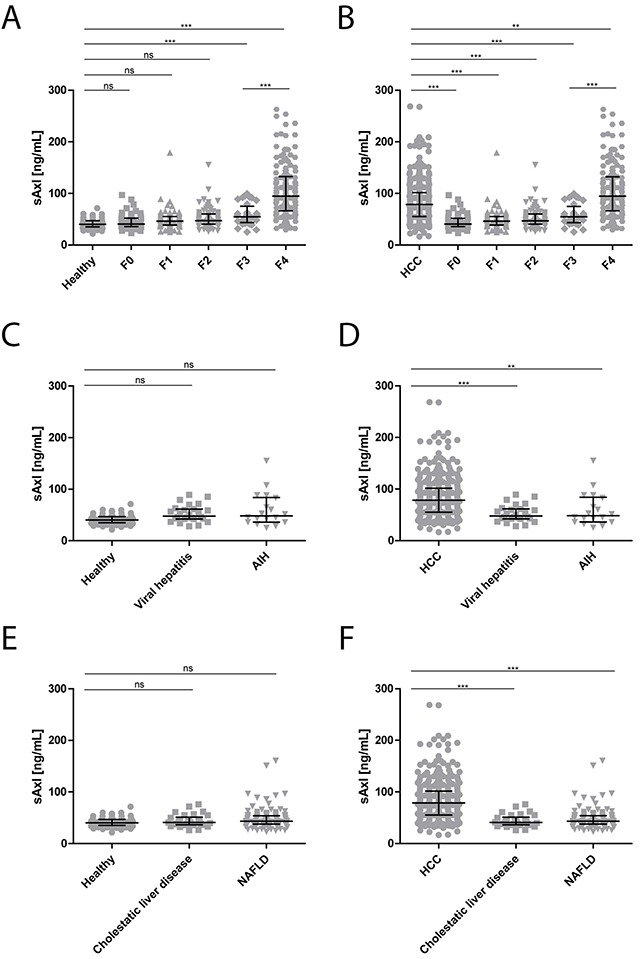
sAxl levels in fibrosis and CLDs **(A)** sAxl serum concentrations in healthy controls (*n* = 75) compared to patients without fibrosis (F0: *n* = 54), with mild fibrosis (F1: *n* = 75), moderate fibrosis (F2: *n* = 80), advanced fibrosis (F3: *n* = 36) and cirrhosis (F4: *n* = 155). **(B)** sAxl serum levels of all HCC patients (*n* = 347) versus fibrosis and cirrhosis patients. **(C)** sAxl serum concentrations in healthy controls (*n* = 75) compared to patients with viral hepatitis (*n* = 26), and autoimmune hepatitis (AIH: *n* = 18). **(D)** sAxl serum levels of all HCC patients versus viral hepatitis and AIH patients. **(E)** Analysis of sAxl serum concentrations in healthy controls (*n* = 75), cholestatic liver disease (*n* = 30) and patients with non-alcoholic fatty liver disease (NAFLD; *n* = 116). **(F)** Statistical comparison between all HCC patients (*n* = 347) to cholestatic liver disease patients and NAFLD. Serum samples were diluted with LowCross-buffer^®^ (Candor, Germany) 1:200 and analyzed for sAxl levels by ELISA. Horizontal bars indicate median levels with interquartile ranges. Statistical significances of the differences between groups were evaluated with Mann-Whitney U test. Ns: not significant. ** *p* ≤ 0.01, *** *p* ≤ 0.001.

We next compared median sAxl levels according to the stage of fibrosis (fibrosis [F] grades 0-4). We could observe significantly increased sAxl levels in advanced fibrosis (F3: 54.67 ng/mL) and cirrhosis (F4: 94.74 ng/mL) when compared to healthy controls (40.15 ng/mL), no fibrosis (F0: 40.70 ng/mL), mild (F1: 46.20 ng/mL) or moderate fibrosis (F2: 46.76 ng/mL) (Figure 2A, Table 2). Interestingly, the cirrhosis patients showed significantly enhanced sAxl levels as compared to advanced fibrosis (Figure 2A and 2B). Notably, all fibrosis stages were significantly lower than HCC, however, the cirrhosis group showed increased sAxl values, respectively (Figure 2B, Table 2). Furthermore, HCC without cirrhosis (61.92 ng/mL) showed higher levels of sAxl as compared to F3 fibrosis (54.67 ng/mL), yet without statistical significance (Table 2).

**Figure 3:**
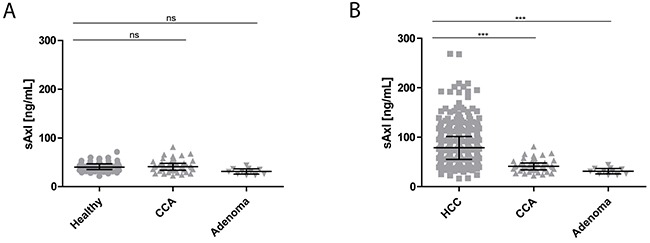
sAxl levels in CCA and liver adenoma patients **(A)** Analysis of sAxl serum concentrations in healthy controls (*n* = 75), CCA patients (*n* = 44) and patients with benign liver adenoma (*n* = 11). **(B)** Statistical comparison between all HCC patients to CCA and adenoma patients. Serum samples were diluted with LowCross-buffer^®^ (Candor, Germany) 1:200 and analyzed for sAxl levels by ELISA. Horizontal bars indicate median levels with interquartile ranges. Statistical significances of the differences between groups were evaluated with Mann-Whitney U test. Ns: not significant. *** *p* ≤ 0.001.

Moreover, we examined sAxl levels in patients with CLD of different etiology. Notably, analysis of patient sera from non-cirrhotic viral hepatitis (chronic hepatitis B or C; 47.87 ng/mL), autoimmune hepatitis (AIH, 48.40 ng/mL), cholestatic liver disease (40.97 ng/mL) and non-alcoholic fatty liver disease (NAFLD; 43.15 ng/mL) showed median concentrations of sAxl similar to healthy controls, but displayed significant differences to HCC patient samples (Figure 2C-2F, Table 2). Importantly, no significant changes in serum sAxl were detected in cholangiocarcinoma (CCA; 41.02 ng/mL) and liver adenoma (31.41 ng/mL) patients versus healthy controls. Additionally, these results were significantly different to sAxl levels in patients with HCC (Figure 3A and 3B, Table 2). Together, these data revealed that patients with CLD, independent of etiology, fail to show increased sAxl levels in the absence of advanced fibrosis/cirrhosis, while large cohorts of patients with F3/F4 fibrosis display elevated levels of sAxl. Liver tumors such as benign liver adenomas or CCA show no alterations in sAxl levels.

### Cut-off levels of sAxl in detecting HCC and cirrhosis

**Figure 4:**
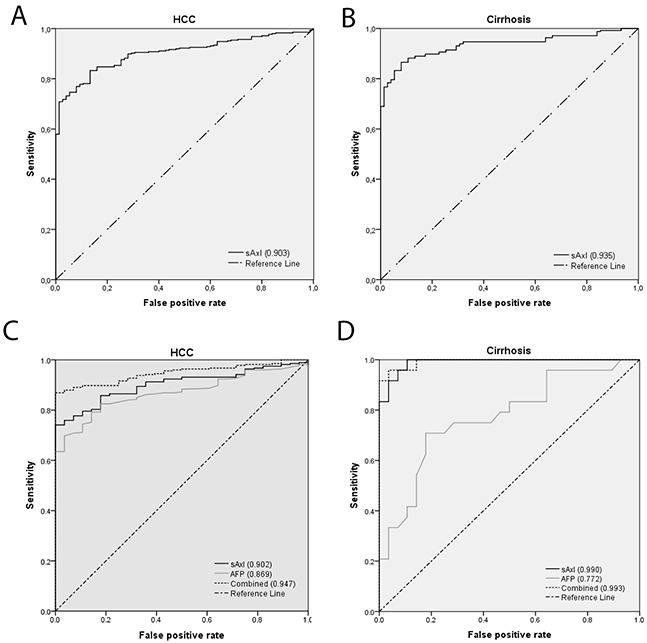
Detection of HCC and cirrhosis by sAxl **(A)** ROC curve of sAxl in healthy controls (*n* = 75) versus HCC (*n* = 347). **(B)** ROC curve of sAxl in healthy controls (*n* = 75) versus cirrhosis (F4, cirrhosis w/o HCC; *n* = 155). **(C)** ROC curve of sAxl, AFP and a combination of both in healthy controls (*n* = 28) versus HCC patients (*n* = 274). **(D)** ROC curve of sAxl, AFP and a combination of both in healthy controls (*n* = 28) versus cirrhosis patients (F4, cirrhosis w/o HCC; *n* = 24). Numbers in parentheses represent area under the curve.

Next we investigated the diagnostic value of sAxl in HCC and cirrhosis with and without incorporation of the established serum marker AFP. ROC curve analysis and the area under the curve (AUC) revealed a high diagnostic performance of sAxl for HCC (AUC 0.903) and also cirrhosis (AUC 0.935) (Figure 4A and 4B). In detail, sAxl displayed a sensitivity of 83.3% and specificity of 86.7% in HCC at a cut-off of 49.71 ng/mL. For cirrhosis, sAxl showed a sensitivity of 80.8% and a specificity of 92.0% at a cut-off of 54.0 ng/mL (Table 3). Interestingly, sAxl outperformed AFP in diagnostic performance in both HCC and cirrhosis. In HCC, sAxl exhibited high sensitivity (74.4%) at the optimal cut-off of 60.42 ng/mL compared to AFP (38.8%) at its cut-off of 20 ng/mL. To evaluate the combination of both markers, we calculated the predicted probability by binary logistic regression. The combination of sAxl and AFP, further exceptionally increased the accuracy (0.947) and sensitivity (86.9%), respectively (Figure 4C, Table 3). The specificity for detection of HCC was 100% for each marker alone and in combination (Table 3). Additionally, in cirrhosis sAxl sensitivity was tremendously high (100%) at a cutoff of 54.47 ng/mL. For AFP we could not depict a clinical valuable cutoff, and therefore AFP showed an intermediate sensitivity of 70.8% at a cutoff of 3.65 ng/mL. This cut-off involved a lot of healthy subjects as well. Here, the combination of sAxl and AFP increased the accuracy (0.993) and slightly decreased the sensitivity (95.8%) as compared to sAxl alone (Figure 4D, Table 3).

**Table 3:** Performance of sAxl and AFP in the detection of HCC and chronic liver diseases

	Sample size	AUC (95% CI)	Sensitivity (%)	Specificity (%)	PPV (%)	NPV (%)	Youden's index	Cut-off (ng/mL)
**sAxl**								
**HCC vs. HC**	347 vs. 75							
sAxl		0.903 (0.873 - 0.932)	83.3	86.7	96.7	52.9	0.70	49.71
**HCC with cirrhosis vs. HC**	272 vs. 75							
sAxl		0.929 (0.903 - 0.956)	89.0	86.7	96.0	68.4	0.76	49.71
**HCC w/o cirrhosis vs. HC**	35 vs. 75							
sAxl		0.789 (0.688 - 0.891)	54.3	96.0	86.4	81.8	0.50	58.39
**Cirrhosis vs. HC**	155 vs. 75							
sAxl		0.935 (0.909 - 0.961)	80.8	92.0	97.1	59.5	0.79	54.0
**Fibrosis F3 vs. HC**	36 vs. 75							
sAxl		0.810 (0.687 - 0.933)	72.0	89.3	69.2	90.5	0.61	52.86
**Fibrosis F3, cirrhosis vs. HC**	191 vs. 75							
sAxl		0.918 (0.885 - 0.950)	82.7	92.0	96.3	67.7	0.75	53.89
**sAXL + AFP**								
**HCC vs. HC**	274 vs. 28							
sAxl		0.902 (0.865 - 0.939)	74.4	100.0	100.0	28.0	0.74	60.42
AFP		0.869 (0.825 - 0.914)	38.8	100.0	100.0	14.2	0.39	20.00
sAxl + AFP		0.947 (0.922 - 0.972)	86.9	100.0	100.0	29.5	0.87	
**HCC with cirrhosis vs. HC**	194 vs. 28							
sAxl		0.937 (0.905 - 0.969)	80.4	96.4	99.4	41.5	0.80	60.42
**AFP**		0.855 (0.803 - 0.907)	32.5	100.0	100.0	17.6	0.32	20.00
sAxl + AFP		0.954 (0.928 - 0.980)	84.0	100.0	100.0	47.5	0.89	
**HCC w/o cirrhosis vs. HC**	28 vs. 28							
sAxl		0.804 (0.682 - 0.925)	60.7	96.4	94.4	71.1	0.57	61.07
(Continued)								
AFP		0.828 (0.710 - 0.947)	53.6	100.0	100.0	68.3	0.54	20.00
sAxl + AFP		0.941 (0.882 - 1.000)	85.7	96.4	96.0	87.1	0.82	
**Cirrhosis vs. HC**	24 vs. 28							
sAxl		0.990 (0.972 - 1.000)	100.0	89.3	85.7	100.0	0.89	54.47
AFP		0.772 (0.642 - 0.902)	70.8	82.1	77.3	76.7	0.53	3.65
sAxl + AFP		0.993 (0.978 - 1.000)	95.8	96.4	95.8	96.4	0.92	
**sAxl center specific**								
**Vienna**								
HCC vs. HC	40 vs. 75	0.781 (0.682 - 0.880)	77.5	72.0	59.6	85.7	0.50	43.88
Cirrhosis vs. HC	48 vs. 75	0.855 (0.778 - 0.931)	66.7	94.7	88.9	81.6	0.61	56.66
**Graz**								
HCC vs. HC	75 vs. 75	0.885 (0.826 - 0.943)	82.7	86.7	86.1	83.3	0.69	49.78
Cirrhosis vs. HC	90 vs. 75	0.987 (0.968 - 1.000)	97.8	94.7	95.7	97.3	0.92	56.66
**Heidelberg**								
HCC vs. HC	119 vs. 75	0.925 (0.887 - 0.963)	79.0	98.7	99.0	74.8	0.78	60.42
**Hannover**								
HCC vs. HC	20 vs. 75	0.932 (0.862 - 1.000)	80.0	97.3	88.9	94.8	0.77	59.88
Cirrhosis vs. HC	4 vs. 75	0.977 (0.937 - 1.000)	100.0	92.0	40.0	100.0	0.92	55.09
**Ogaki**								
HCC vs. HC	93 vs. 75	0.934 (0.898 - 0.971)	81.7	94.7	95.0	80.7	0.76	56.87

AUC: area under the curve; CI: confidence interval; PPV: positive predictive value; NPV: negative predictive value; HCC: hepatocellular carcinoma; HC: healthy controls. Diagnostic cut-off for AFP in HCC was 20 ng/mL.

Next, we assessed the diagnostic probabilities of sAxl in detecting HCC with and without cirrhosis, advanced fibrosis (F3) alone and in combination with cirrhosis. For HCC with cirrhosis, we obtained a remarkable AUC of 0.929 accompanied by a high sensitivity of 89.0% and specificity of 86.7% (Supplementary Figure 2A, Table 3). Single and combinational assessment of sAxl and AFP disclosed a better AUC of 0.937 for sAxl then for AFP (0.855) in HCC patients with cirrhosis. Also the sensitivity was tremendously better for sAxl (80.4%) as compared to AFP (32.5%). Again, the combination of both markers further increased the AUC (0.954), the sensitivity (84.0%) and specificity (100%) (Supplementary Figure 2B, Table 3). By evaluating the results of HCC patients without liver cirrhosis, we were able to reach an accuracy of 0.789 with a sensitivity of 54.3% and a specificity of 96.0% (Supplementary Figure 2C, Table 3). Results of HCC without cirrhosis including both markers revealed a better AUC for AFP (0.828) as compared to sAxl (0.804) alone, but sAxl showed higher sensitivity (60.7%) and specificity (96.4%) than AFP alone (sensitivity: 53.6%; specificity 100%). Remarkably, the combination of both markers revealed a great accuracy (0.941), sensitivity (85.7%) and specificity (96.4%) of detecting non-cirrhotic HCC (Supplementary Figure 2D, Table 3). Similar results were obtained for advanced fibrosis with an AUC of 0.810, sensitivity of 72.0% and specificity of 89.3% (Supplementary Figure 2E, Table 3). By pooling the results of patients suffering on either advanced fibrosis (F3) or cirrhosis, we reached an AUC of 0.918 resulting in a sensitivity of 82.7% and a specificity of 92.0% compared to advanced fibrosis alone (AUC 0.810) (Supplementary Figure 2E and 2F, Table 3). Overall, these results confirmed the assumption that cirrhosis plays an important role on sAxl levels in serum. The diagnostic performance of sAxl in detecting HCC (Vienna, AUC 0.781; Graz, AUC 0.885; Heidelberg, AUC 0.925; Hannover, AUC 0.932; Ogaki, AUC 0.934) and cirrhosis (Vienna, AUC 0.855; Graz, AUC 0.987; Hannover, AUC 0.977) was high across all centers included in this study (Supplementary Figures 3 and 4, Table 3).

### Cellular sources of sAxl during advanced fibrosis and cirrhosis

**Figure 5:**
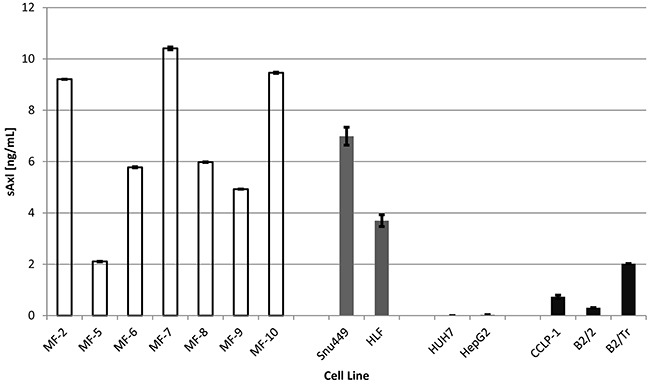
sAxl release of liver myofibroblasts, mesenchymal and epithelial hepatoma cell lines and CCA cell lines Evaluation of total sAxl release of seven liver myofibroblast cell lines (white), two mesenchymal hepatoma cell lines (dark gray), two epithelial hepatoma cell lines (gray) and three cholangiocellular carcinoma cell lines (black) by ELISA. Data are expressed as mean ± s.d.

As recently reported, we showed that mesenchymal HCC cell lines release high levels of sAxl whereas epithelial HCC cell lines fail to release sAxl [23]. Here, we confirmed these data, and additionally found that CCA cell lines show only modest release of sAxl, which is in accordance with low sAxl levels in sera of CCA patients (Figure 3A and 3B). Furthermore, we investigated the sAxl release of liver myofibroblasts and observed a strong sAxl release by most of these cell lines which are generally associated with fibrosis progression (Figure 5) [25–28]. These data suggest that sAxl is predominantly released by myofibroblasts which accumulate during increased fibrosis.

## DISCUSSION

The present study evaluated the diagnostic performance of sAxl for the detection of early HCC and patients at high risk of HCC in a large multicentric cohort of 1067 patients. In comparison with healthy controls, sAxl showed a high diagnostic accuracy for the detection of HCC in patients with (AUC 0.929) and without cirrhosis (AUC 0.789). However, sAxl was additionally identified as an excellent marker of advanced fibrosis (F3; AUC 0.810) and cirrhosis (F4; AUC 0.935). Diagnostic accuracy was further improved, if both sAxl and AFP were combined (HCC with cirrhosis: AUC 0.954; HCC w/o cirrhosis: AUC 0.941; cirrhosis: AUC 0.993).

Recently, we reported a high accuracy of sAxl in the diagnosis of early as well as advanced stages of HCC [24]. The diagnostic value of sAxl for early HCC (BCLC A) and all other BCLC stages of HCC was confirmed in the present study (Figure 1B). sAxl levels were BCLC stage-dependent, since patients with BCLC stage D showed significantly elevated sAxl levels in comparison to BCLC A, B, or C, and healthy controls. Of note, sAxl was not only significantly elevated in patients with HCC and cirrhosis in the present study, but also in patients with HCC without cirrhosis (Figure 1A) as compared to healthy controls. This may lead to the assumption, that sAxl accurately detects HCC, both in the presence as well as in the absence of liver cirrhosis. However, no significant difference in sAxl levels was found when (i) patients with HCC and cirrhosis were compared to patients with HCC but without cirrhosis or cirrhosis without HCC (Supplementary Figure 5), and (ii), when HCC patients without cirrhosis were compared to patients with CLD and fibrosis grade 3 without HCC. Yet, sAxl levels in fibrosis grades 0 to 2 were similar to levels in healthy controls. This means that sAxl is an excellent marker for advanced fibrosis or liver cirrhosis independently of the presence or absence of HCC.

This is contradictory to our previous published data showing a significant difference between 26 patients with very early HCC (BCLC 0) and 30 patients with liver cirrhosis [24]. In the present study, 155 patients with cirrhosis from different centers were included in the study and compared to 272 HCC patients with cirrhosis and 35 HCC patients without cirrhosis. Therefore, the divergent findings may result from a significantly lower case number in our previous study. A possible explanation for our results is that liver fibrosis, mainly characterized by the accumulation of extracellular matrix components derived from activated hepatic stellate cells (HSC), promotes an environment permissive for tumor development. HSCs transform during chronic liver injury from a quiescent state into a myofibroblast-like phenotype, which proliferate and migrate towards areas of necrosis and regeneration [29]. Our findings are also in line with a study recently published by Barcena at el showing that sAxl is involved in liver fibrosis progression [25]. In this study using HSCs obtained from wildtype and Axl^−/−^ mice treated with recombinant Gas6, Axl siRNAs or the Axl inhibitor BGB324, the Gas6/Axl axis was reported to be required for HSC activation. Furthermore, Axl serum levels increased in parallel to CLD progression in line with our data demonstrating that sAxl levels are rising with increasing fibrosis stages (Figure 2A and 2B, Table 2). This was independent of liver disease etiology (Figure 2C-2F).

Our findings are further supported by the fact that 6 out of 7 liver myofibroblast cell lines under investigation showed a strong sAxl release, suggesting that sAxl is predominantly released by myofibroblasts during fibrosis progression (Figure 5). This may be explained by an increased proteolysis of Axl in myofibroblasts during fibrosis development, facilitating an HCC permissive state [30]. Moreover, sAxl was specific for HCC and liver fibrosis progression in all tested scenarios as sAxl was significantly elevated in HCC, but not in CLD without HCC, such as chronic viral hepatitis, AIH, cholestatic liver disease, and NAFLD (Figure 2C-2F). Additionally, sAxl levels in CCA and liver adenomas remained as low as in healthy controls (Figure 3A), and were therefore significantly lower than in HCC patients (Figure 3B). As we reported in a previous manuscript, sAxl is also significantly higher in HCC than in other solid tumors, such as ovarian, breast and hepatic metastases of CRC [24].

Based on our present study, sAxl has an excellent diagnostic accuracy for the detection of advanced fibrosis/cirrhosis (AUC 0.918) and HCC at all stages (AUC 0.929) superior to AFP as well as has a favourable accuracy in HCC without cirrhosis (AUC 0.789) (Table 3). Nevertheless, measurement of sAxl alone may not enable clinicians to securely differentiate between advanced fibrosis/cirrhosis and early HCC.

Combining both sAxl and AFP, however, may further increase diagnostic accuracy. Recently, Jang et al compared the diagnostic accuracy of 4 HCC biomarkers alone and in combination (AFP, osteopontin, DKK-1 and protein induced by vitamin K absence (PIVKA-II)) in 208 HCC patients and 193 liver cirrhotic control patients from Korea [31]. Of all tested biomarkers, AFP performed best for the detection of early HCC at a cut-off of 20 ng/mL (AUC: 0.678, sensitivity: 45.2%, specificity: 90.2%). The best performing combination of biomarkers was AFP plus DKK-1 (cut-off: 500 pg/mL) reaching an AUC of 0.693 (sensitivity: 66.0%, specificity: 72.5%). Further studies investigated the combination of annexin A2 and AFP (combined AUC 0.83) or thioredoxin and AFP (combined AUC 0.88) achieving even higher diagnostic accuracy [15, 32, 33]. In our study, the combination of AFP (cut-off of ≥ 20 ng/mL) and sAxl at a cutoff of ≥ 61 ng/mL reached excellent diagnostic accuracy with a sensitivity and specificity of 84.0% and 100% for HCC with cirrhosis (AUC 0.954), and 85.7% and 96.4% for HCC without cirrhosis (AUC 0.941), respectively. Interestingly, our study further revealed a high diagnostic accuracy for the detection of HCC even in AFP-negative cases (AUC=0.790; sensitivity 71.0% and specificity 83.0%) when comparing all AFP-negative HCC patients (cut-off < 20 ng/mL AFP) with AFP-negative fibrosis/cirrhosis (F1-F4) patients (Supplementary Figure 6).

During the last update of the clinical practice guidelines 2012 of the European Association for the Study of the Liver, AFP was abandoned and diagnostic criteria are still based on imaging techniques and/or histopathology [7]. Nevertheless, many centers practice regular AFP measurements complementing imaging studies for HCC surveillance in cirrhotic patients. AFP, however, can only be taken into consideration in case it is elevated or shows an increase over time.

Owing to the large sample size and multicenter approach, detailed clinical data of patients were not available. Although our analysis includes a large cohort of study participants, patient numbers available for subgroup analysis are rather low and especially data on BCLC stages, different CLD and combination of AFP with sAxl should be confirmed in further series. In addition, our study cohort represents a cross sectional patient collective so that data on longitudinal courses of sAxl and AFP levels cannot be provided. In conclusion, sAxl is an excellent marker for advanced liver fibrosis/cirrhosis in most common CLDs and is specific for HCC. Used in combination with AFP, sAxl shows a high potential as an accurate surveillance marker in patients at high risk for HCC.

## MATERIALS AND METHODS

### Study population

Serum samples from patients with HCC (*n* = 347), intrahepatic CCA (*n* = 44), liver adenoma (*n* = 11), liver fibrosis (*n* = 245), liver cirrhosis (*n* = 155), and CLDs (*n* = 190) such as chronic infection with hepatitis B and hepatitis C virus (viral hepatitis), autoimmune hepatitis (AIH), primary biliary cirrhosis and primary sclerosing cholangitis (cholestatic liver disease) or non-alcoholic fatty liver disease (NAFLD) were collected from different centers in Europe and Asia. In addition, age-matched serum samples from healthy controls (*n* = 75) were included in the analysis. Samples were collected at the Medical University of Vienna (MUW) (Vienna, Austria; *n* = 416), Medical University Graz (Graz, Austria; *n* = 335), University of Regensburg Hospital (Regensburg, Germany; *n* = 21), University Hospital Heidelberg (Heidelberg, Germany; *n* = 119), Hannover Medical School (Hannover, Germany; *n* = 83) and at WAKO Pure Chemical Industries (Ogaki, Japan; *n* = 93) (Supplementary Figure 1). Available AFP data was ascertained at time of sample drawing for sAxl analysis and was available in *n* = 298 patients and *n* = 28 healthy subjects. All serum samples were collected prior to therapeutic intervention. All blood samples were centrifuged to prepare sera that were free of cellular components and stored at -80°C until further processing.

In patients in whom liver biopsy was performed due to routine diagnostics, liver tissue samples were reviewed by two independent pathologists to confirm the stage of fibrosis in each sample. Fibrosis grading was performed according to METAVIR as appropriate (no fibrosis (F0), mild fibrosis (F1), moderate fibrosis (F2), advanced fibrosis (F3), or cirrhosis (F4)). HCCs were diagnosed according to non-invasive imaging criteria or by histology [7]. CCA was diagnosed based on histology.

The study was conducted in compliance with the Declaration of Helsinki and was approved by the responsible Ethical Committees of the study centers. Informed consent was obtained from all study participants prior to the performance of study related procedures. CLD was diagnosed by laboratory tests as well as by imaging studies such as US, magnetic resonance imaging, or computed tomography according to international standards. Healthy controls were recruited using a database of registered healthy controls. Subjects with alterations in liver serology or incomplete data, as well as other malignancies were excluded from the study (Supplementary Figure 1). Further clinical information of HCC patients concerning age, sex, hepatitis B virus (HBV) and hepatitis C virus (HCV) status, as well as cirrhosis, vascular invasion and lymph node metastasis was partially available (Table 1). Subjects were stratified on the basis of underlying liver disease, tumor staging and available AFP levels (Table 2). A cohort of HCC patients could be further classified by the Barcelona Clinic Liver Cancer (BCLC) classification into early (BCLC stage A; *n* = 45), intermediate (BCLC stage B; *n* = 45), advanced (BCLC stage C; *n* = 67) and end-stage HCC (BCLC stage D; *n* = 12).

### Enzyme-linked immunosorbent assay

Solid phase sandwich ELISAs were carried out according to manufacturer's protocol of ‘human Axl DuoSet^®^ ELISAs’ (R&D Systems, Minneapolis, USA) including modifications for optimization of the assay [34]. These modifications resulted in higher detectable serum levels of sAxl as compared to our recent study [24]. Notably, these variations of sAxl concentrations in the serum were found to depend on the dilution medium and the factor of dilution [34]. Samples and detection antibody were diluted in LowCross-buffer^®^ (Candor Bioscience, Wangen, Germany) instead of using Dulbecco's phosphate buffered saline (DPBS) containing 1% bovine serum albumin (BSA). Briefly, a microtiter plate (96 well, high binding; Greiner Bio-One, Frickenhausen, Germany) was coated overnight at room temperature (RT) with 2 μg/mL human Axl capture antibody diluted 1:180 in DPBS (PromoCell, Heidelberg, Germany). Afterwards, 3 washing steps with each 300 μL of DPBST, containing 0.05% Tween^®^ 20 (AppliChem, Darmstadt, Germany) were carried out by utilizing a HydroSpeed™ plate washer (Tecan, Männedorf, Switzerland). The plate was blocked with 300 μL reagent diluent, phosphate buffered saline (PBS) containing 1% high grade BSA (Probumin^®^, Millipore, Illinois, USA), for 2 hours at RT. The washing steps were repeated and subsequently, 100 μL of recombinant Axl standards and samples, diluted 1:200, were added to the 96 well plates and incubated for 2 hours at RT. Mentioned washing steps were repeated followed by addition of 100 μL of 50 ng/mL human Axl biotinylated detection antibody, diluted 1:180 in LowCross-buffer^®^ (Candor Bioscience, Wangen, Germany), to each well. After incubation for 2 hours at RT, the plates were washed again three times and 100 μL streptavidin-horseradish peroxidase working dilution was added per well. Dark incubation at RT for 20 minutes and three times washing with PBST prepared the plate for incubation with 100 μL of a 1:1 mixture of hydrogen peroxidase and tetramethylbenzidine (Substrate A and B, R&D Systems Minneapolis, USA) for further 15 minutes in the dark. Appearing enzymatic color reaction was finally stopped with 50 μL of 2 N sulfuric acid (Carl Roth, Karlsruhe, Germany). Subsequently, the optical density (OD) was determined at 450 nm using a microplate reader (Biochrom, Berlin, Germany) with a wavelength correction at 620 nm to correct for optical imperfections in the plate. On each plate, a separate seven-point standard curve was established with absorbance on the vertical axis and the concentrations on the horizontal axis. The serial dilutions of the recombinant Axl standard curve included 4.000, 2.000, 1.000, 500, 250, 125 and 62.5 pg/mL. LowCross-buffer^®^ was used as a negative control. All samples were measured in biological replicates and technical triplicates.

### Statistical analysis

Microsoft Excel 2010 (Microsoft, Redmond, USA), GraphPad Prism 5 (GraphPad Software, La Jolla, USA) in combination with IBM SPSS software v 20.0 (IBM Corp., Armonk, USA) was utilized for statistical analysis and continuous data comparison. Nonparametric, two-sided Mann-Whitney U tests were carried out for single comparisons. *p* values < 0.05, P < 0.01, or P < 0.001 were considered statistically significant.

### Receiver operating characteristics

IBM SPSS software v 20.0 (IBM Corp., Armonk, USA) was used to plot sensitivity against false positive rate to generate receiver operating characteristics (ROC) curves. ROC curves were compiled using either sAxl or AFP alone or in combination. For evaluation of both biomarkers, a combinatorial variable was generated by binary logistic regression through an iterative maximum likelihood procedure. The diagnostic performance was evaluated by ROC curve analysis considering the area under the curve (AUC) with a 95% confidence interval (CI). Youden Index (*J*) calculation led to optimal cut-off values for sAxl selected at the concentrations representing the highest sum of sensitivity and specificity, respectively. In the case of AFP, the clinically established cutoff value of 20 ng/mL was applied [13, 35–38].

### Cell culture

The hepatic myofibroblast cell lines MF-2, MF-5, MF-6, MF-7, MF-8, MF-9, and MF-10 [39], the mesenchymal and epithelial HCC cell lines (SNU449, HLF and HUH7, HepG2, respectively), as well as the CCA cell lines CCLP-1, B2/2 and B2/Tr [40] were cultured with appropriate ACL and RPMI media plus 10% fetal calf serum (FCS). B2/2 and B2/Tr cells were isolated from biopsies of CCA patients. Cells were authenticated by short tandem repeat analysis in July 2016. All cells were routinely screened for the absence of mycoplasma. After cells reached a confluency of about 80%, the FCS-rich media was removed and 1.5 mL serum-free RPMI was added to each cell line. Subsequently, cells were allowed to release sAxl into supernatants for 24 hours. Next, the supernatants were cleared from cell debris and analyzed for sAxl levels by ELISA. Finally, the cells were counted (CASY^®^, OLS OMNI Life Science, Bremen, Germany) after harvesting of cell supernatants to allow normalization of sAxl values to cell numbers.

## SUPPLEMENTARY FIGURES


