# Supplementary material for: Soluble Axl is an accurate biomarker of cirrhosis and hepatocellular carcinoma development: results from a large scale multicenter analysis
Source: Oncotarget. 2017 May 3;8(28):46234–48. doi: 10.18632/oncotarget.17598 (PMC5542263; doi:10.18632/oncotarget.17598)
Supplement: Supplementary file 1 [file oncotarget-08-46234-s001.pdf]

Soluble Axl is an accurate biomarker of cirrhosis and hepatocellular carcinoma development: results from a large scale multicenter analysis

SUPPLEMENTARY MATERIALS

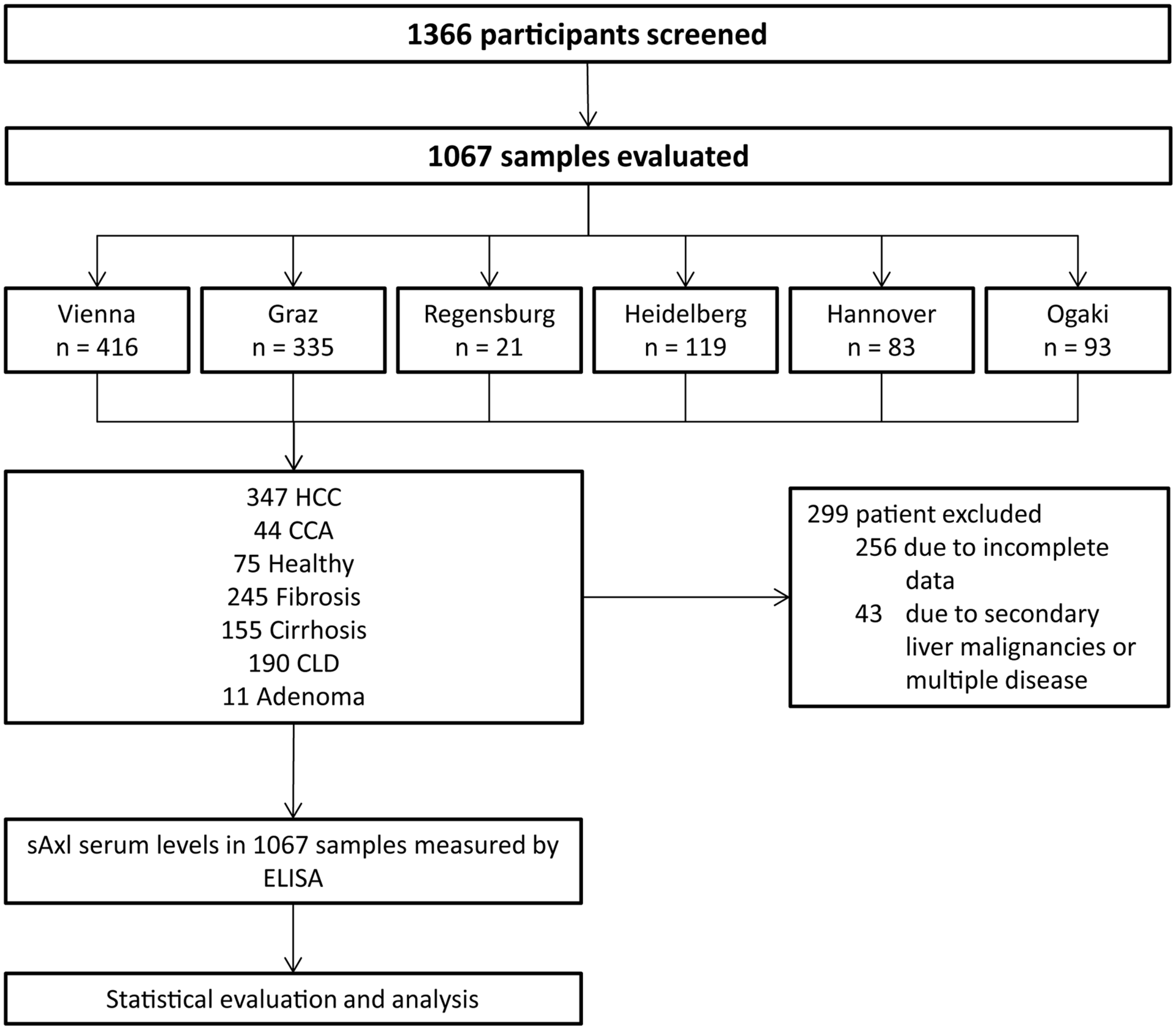

Supplementary Figure 1: Study design. ELISA: enzyme-linked immunosorbent assay; HCC: hepatocellular carcinoma; CCA: cholangiocarcinoma; CLD: chronic liver disease.

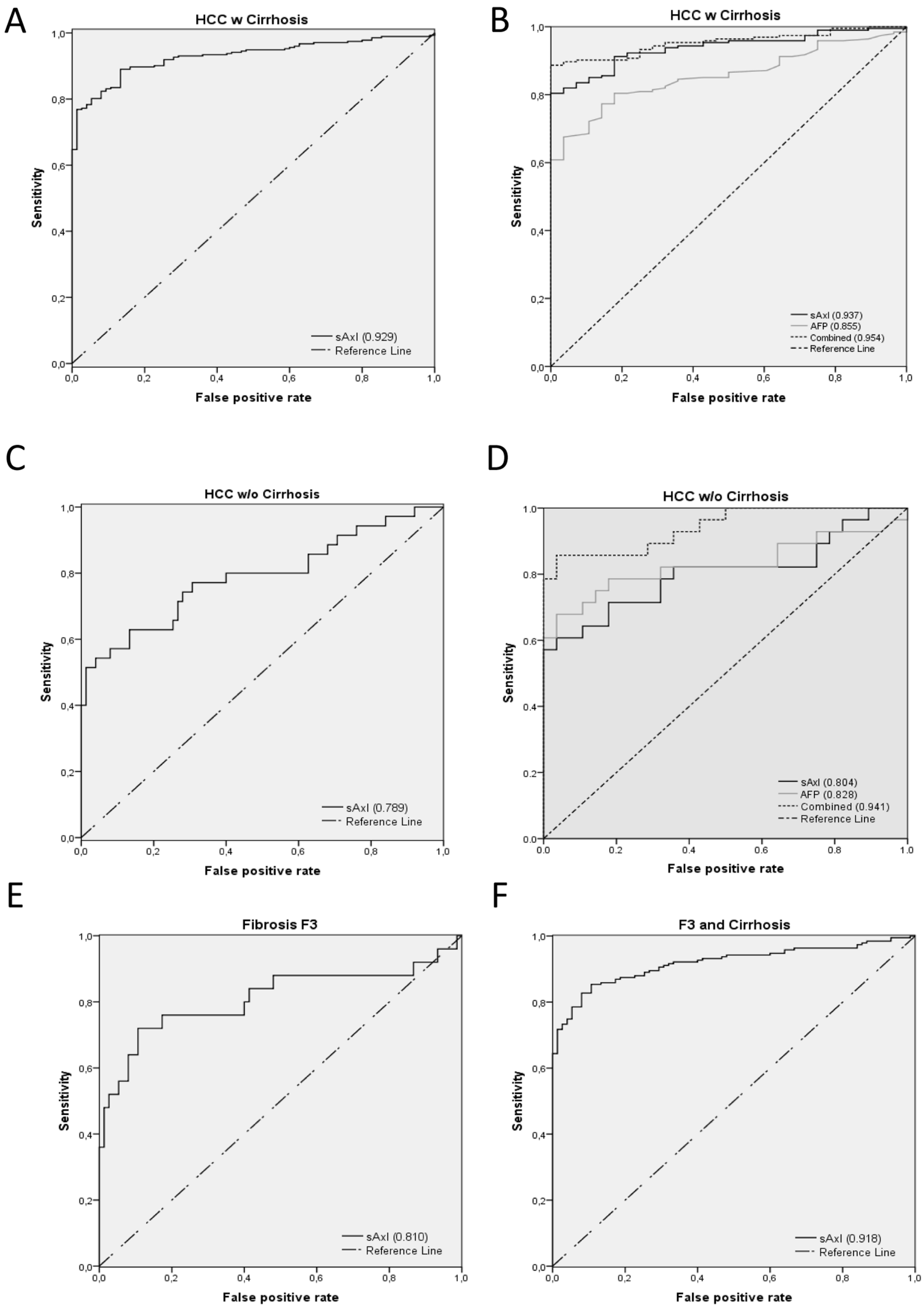

Supplementary Figure 2: Detection of HCC without cirrhosis and advanced fibrosis by sAxl. (A) ROC curve of sAxl in healthy controls (n = 75) versus HCC patients with cirrhosis (n = 272). **(B)** ROC curve of sAxl, AFP and a combination of both in healthy controls (n = 28) versus HCC patients with cirrhosis (n = 194). **(C)** ROC curve of sAxl in healthy controls (n = 75) versus HCC patients without cirrhosis (n = 35). **(D)** ROC curve of sAxl, AFP and a combination of both in healthy controls (n = 28) versus HCC patients without cirrhosis (n = 28). **(E)** ROC curve of sAxl in healthy controls (n = 75) versus advanced fibrosis patients (F3: n = 36). **(F)** ROC curve of sAxl in healthy controls (n = 75) versus a combination of advanced fibrosis and cirrhosis patients (n = 191). Numbers in parentheses represent area under the curve.

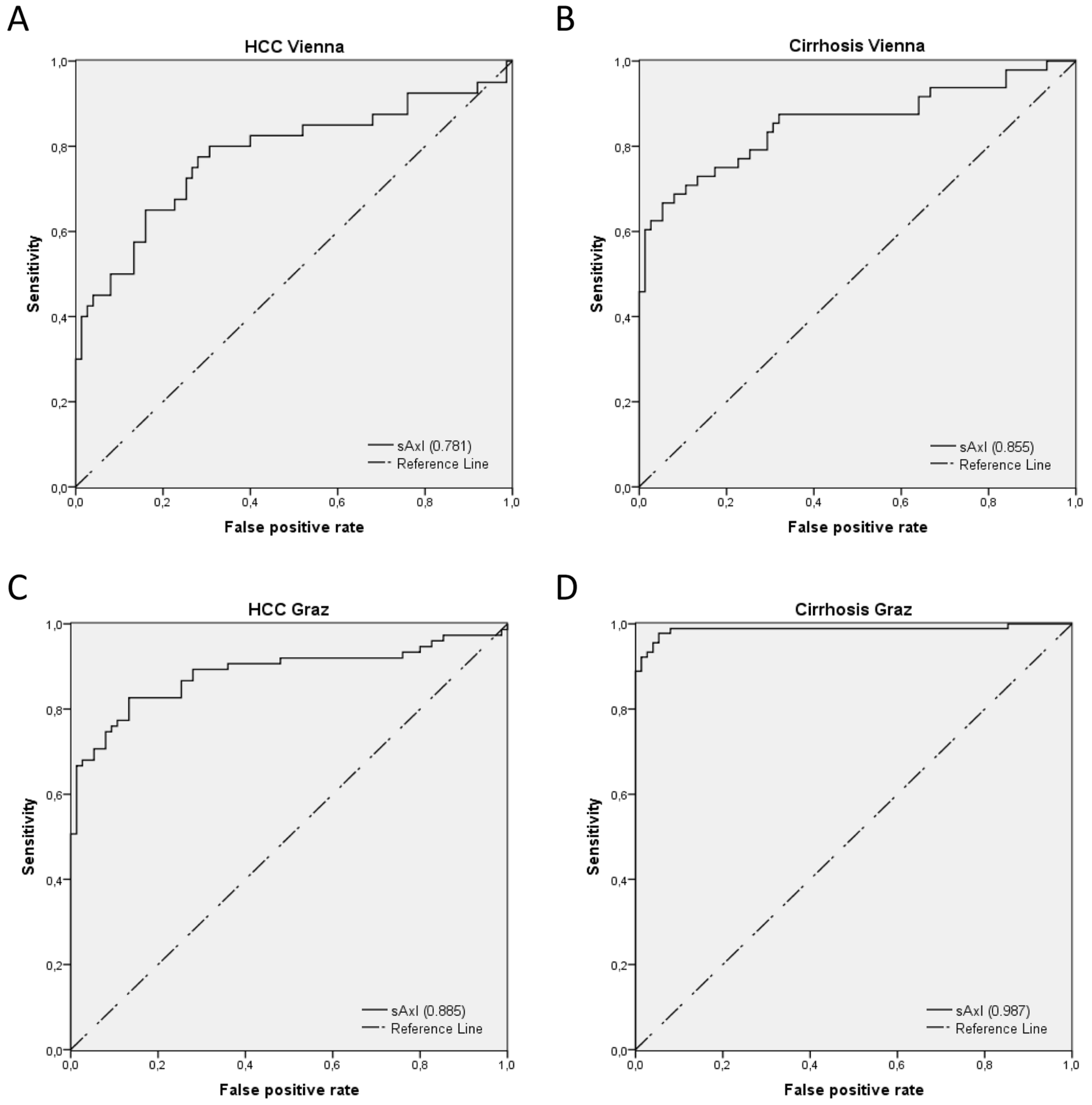

Supplementary Figure 3: Center-specific detection of HCC and cirrhosis by sAxl. **(A)** ROC curve of sAxl in healthy controls (n = 75) versus HCC patients (Vienna: n = 40). **(B)** ROC curve of sAxl in healthy controls (n = 75) versus cirrhosis patients (F4, cirrhosis w/o HCC; Vienna: n = 48). **(C)** ROC curve of sAxl in healthy controls (n = 75) versus HCC patients (Graz: n = 75). **(D)** ROC curve of sAxl in healthy controls (n = 75) versus cirrhosis patients (F4, cirrhosis w/o HCC; Graz: n = 90). Numbers in parentheses represent area under the curve.

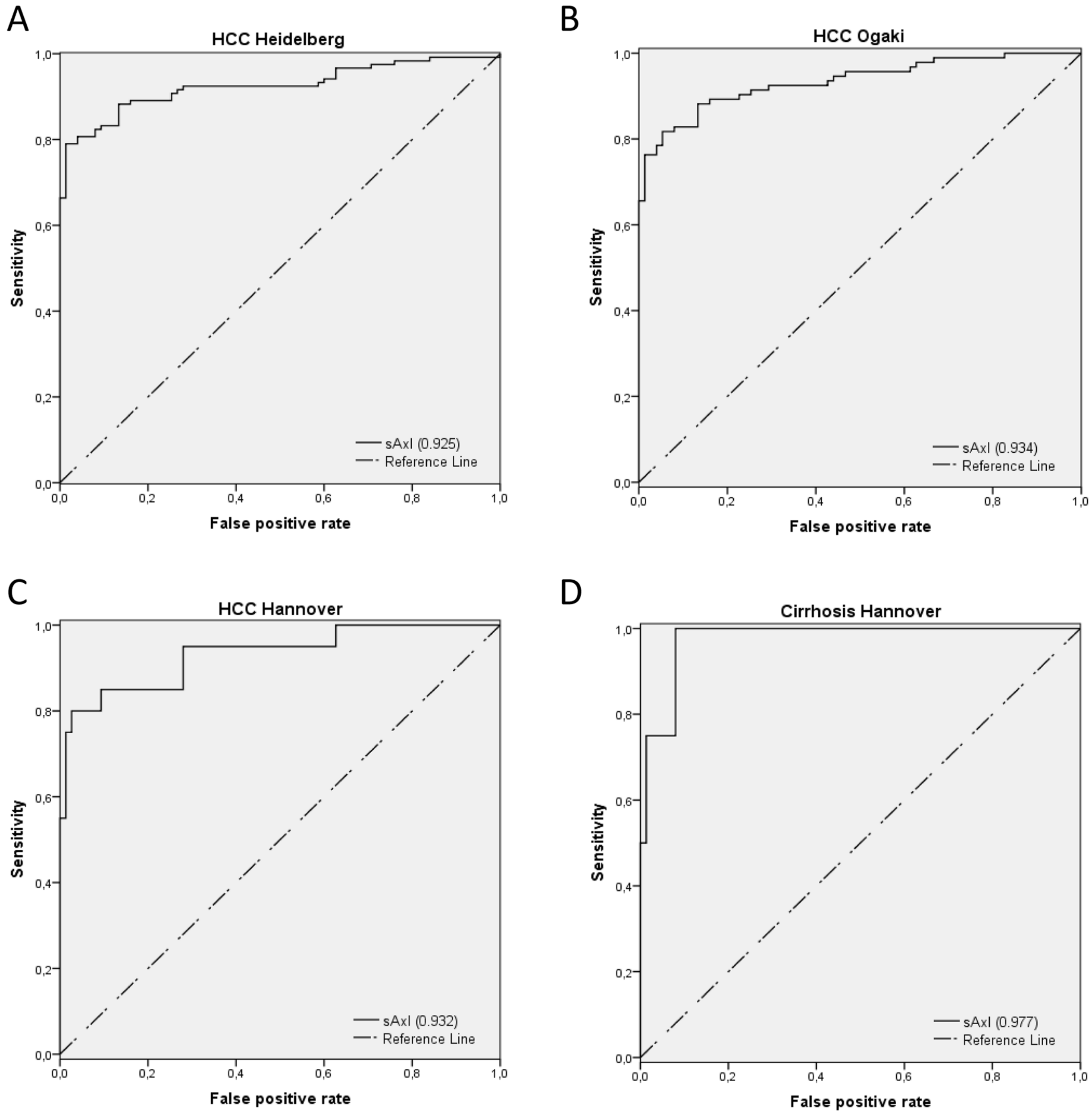

Supplementary Figure 4: Center-specific detection of HCC and cirrhosis by sAxl. **(A)** ROC curve of sAxl in healthy controls (*n* = 75) versus HCC patients (Heidelberg: *n* = 119). **(B)** ROC curve of sAxl in healthy controls (*n* = 75) versus HCC patients (Ogaki: *n* = 93). **(C)** ROC curve of sAxl in healthy controls (*n* = 75) versus HCC patients (Hannover: *n* = 20). **(D)** ROC curve of sAxl in healthy controls (*n* = 75) versus cirrhosis patients (F4, cirrhosis w/o HCC; Hannover: *n* = 4). Numbers in parentheses represent area under the curve.

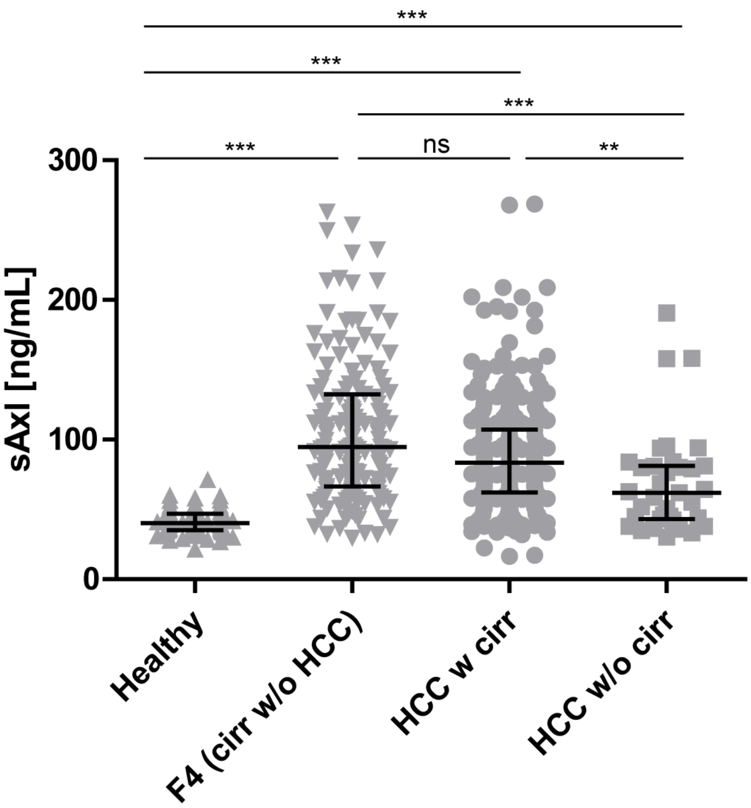

Supplementary Figure 5: sAxl levels in HCC patients with and without cirrhosis compared to cirrhosis and healthy control. Analysis of sAxl serum concentrations in healthy controls (*n* = 75), cirrhosis patients (F4, cirrhosis w/o HCC; *n* = 155), HCC patients with cirrhosis (*n* = 272) and HCC patients without cirrhosis (*n* = 35). Serum samples were diluted with LowCross-buffer® (Candor, Germany) 1:200 and analyzed for sAxl levels by ELISA. Horizontal bars indicate median levels with interquartile ranges. Statistical significances of the differences between groups were evaluated with Mann-Whitney U test. Ns: not significant. ** = $p \leq 0.01$, *** = $p \leq 0.001$.

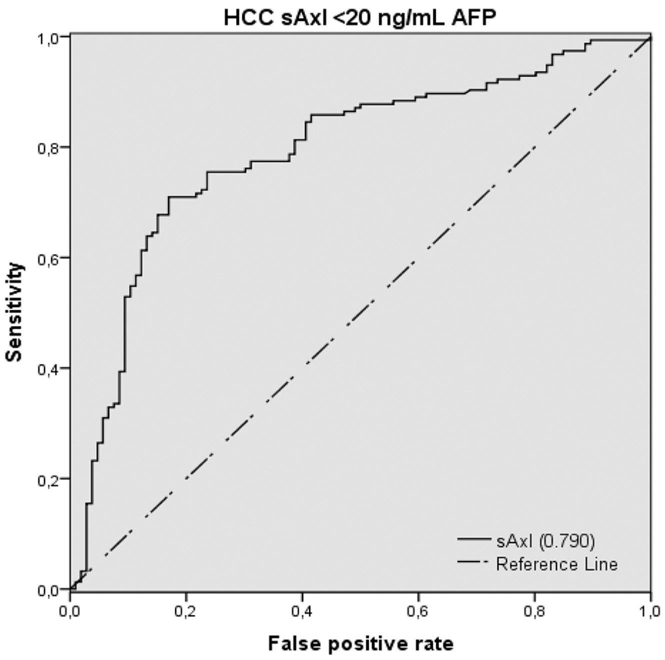

Supplementary Figure 6: Detection of AFP-negative HCC and AFP-negative fibrosis/cirrhosis (F1 - F4) by sAxl. ROC curve of sAxl in AFP-negative (<20 ng/mL) HCC patients (n = 155) versus AFP-negative fibrosis and cirrhosis patients (n = 106). The number in parentheses represents the area under the curve AUC (0.95% CI): 0.709 (0.733 – 0.847) with a detection sensitivity of 71.0% and a specificity of 83.0% at a Youden's index of 0.54 and a cut-off of 61.36 ng/mL serum concentration. PPV and NPV calculations resulted in 85.2% and 66.2%, respectively. Due to missing space and to avoid further complexity, these data are not shown in Table 3. Statistical significance between the groups was evaluated by Mann-Whitney U test revealing high differential diagnostic power with a p value of <0.0001 (data not shown). Additionally, verified by direct sAxl median comparison of the two groups: AFP negative fibrosis/cirrhosis (80.44 ng/mL) and AFP negative HCC (46.37 ng/mL).
